# Heritability and Genetic Correlations Explained by Common SNPs for Metabolic Syndrome Traits

**DOI:** 10.1371/journal.pgen.1002637

**Published:** 2012-03-29

**Authors:** Shashaank Vattikuti, Juen Guo, Carson C. Chow

**Affiliations:** Laboratory of Biological Modeling, National Institute of Diabetes and Digestive and Kidney Diseases (NIDDK), National Institutes of Health (NIH), Bethesda, Maryland, United States of America; The University of Queensland, Australia

## Abstract

We used a bivariate (multivariate) linear mixed-effects model to estimate the narrow-sense heritability (*h^2^*) and heritability explained by the common SNPs (*h_g_^2^*) for several metabolic syndrome (MetS) traits and the genetic correlation between pairs of traits for the Atherosclerosis Risk in Communities (ARIC) genome-wide association study (GWAS) population. MetS traits included body-mass index (BMI), waist-to-hip ratio (WHR), systolic blood pressure (SBP), fasting glucose (GLU), fasting insulin (INS), fasting trigylcerides (TG), and fasting high-density lipoprotein (HDL). We found the percentage of *h^2^* accounted for by common SNPs to be 58% of *h^2^* for height, 41% for BMI, 46% for WHR, 30% for GLU, 39% for INS, 34% for TG, 25% for HDL, and 80% for SBP. We confirmed prior reports for height and BMI using the ARIC population and independently in the Framingham Heart Study (FHS) population. We demonstrated that the multivariate model supported large genetic correlations between BMI and WHR and between TG and HDL. We also showed that the genetic correlations between the MetS traits are directly proportional to the phenotypic correlations.

## Author Summary

The narrow-sense heritability of a trait such as body-mass index is a measure of the variability of the trait between people that is accounted for by their additive genetic differences. Knowledge of these genetic differences provides insight into biological mechanisms and hence treatments for diseases. Genome-wide association studies (GWAS) survey a large set of genetic markers common to the population. They have identified several single markers that are associated with traits and diseases. However, these markers do not seem to account for all of the known narrow-sense heritability. Here we used a recently developed model to quantify the genetic information contained in GWAS for single traits and shared between traits. We specifically investigated metabolic syndrome traits that are associated with type 2 diabetes and heart disease, and we found that for the majority of these traits much of the previously unaccounted for heritability is contained within common markers surveyed in GWAS. We also computed the genetic correlation between traits, which is a measure of the genetic components shared by traits. We found that the genetic correlation between these traits could be predicted from their phenotypic correlation.

## Introduction

Obesity associated traits such as central adiposity, dyslipidemia, hypertension, and insulin resistance are major risk factors for type 2 diabetes and cardiovascular complications [1]. The constellation of these traits has been termed metabolic syndrome (MetS). Understanding the genetic factors underlying these traits and how they are correlated is clinically important. Large-scale genotyping investigations such as genome-wide association studies (GWAS) are useful tools for identifying genetic factors. However, significant genetic variants discovered in GWAS explain only a small proportion of the expected narrow-sense heritability, *h^2^*, defined as the ratio of additive genetic variance to phenotypic variance [2]. This discrepancy underlies the debate concerning “missing” genetic factors among the common variants [3], [4].

The main approach of GWAS has been to identify significant single-nucleotide polymorphisms (SNPs) by examining each SNP individually for significance. The *h^2^* attributed to that marker is then given by *2f(1−f)a^2^*, where *f* is the frequency of the marker and *a* is the additive effect. To reduce the chance of false positives, a stringent *p*-value criterion has been adopted (typically *p* = 5*10^−8^, based on an adjusted *p*-value of 0.05 for one-million tests). It has been suggested that this selection criterion is too conservative [5] and that some of the missing heritability may be linked to genetic markers of small effect that fail this stringent cutoff.

Alternatively, the narrow sense heritability explained by the common SNPs, *h_g_^2^*, may be estimated by adapting a linear mixed-effects model [6], [7] that is used to estimate *h^2^*. This model decomposes the phenotypic variance into genetic and residual variance components. Usually, the model is applied to related individuals where the genetic relationships are estimated by using family pedigree or genetic markers [8], [9]. Yang et al. [6], [7] pointed out that *h_g_^2^* could be estimated using genetic relationships obtained from the common SNPs for unrelated individuals. The main assumed difference between *h_g_^2^* and *h^2^* is due to the difference in linkage disequilibrium (LD) between the common SNP markers and the rest of the genome, with the assumption that closely related individuals would be in greater LD than unrelated individuals. Thus, heritability estimated with the genetic relationships of unrelated individuals is attributed to the common variants while that estimated with genetic relationships of related individuals is attributed to the entire genome. While the method does not identify single variants, it provides the maximum expected variance expected by the set of markers or the relative complement of the set (e.g., common versus rare variants). Recently, it has been shown that a large proportion of *h^2^* is explained by the common single-nucleotide polymorphisms (SNPs) for several traits using this model [6], [7]. Here, we showed that large proportions of the phenotypic variance for several metabolic syndrome (MetS) traits were also captured by the common SNPs. Among these, we validated the height and body-mass index estimates by Yang et al. [6], [7] in independent GWAS populations. We also quantified the genetic correlation between traits explained by the common SNPs.

## Results

We estimated *h^2^* and *h_g_^2^* for height and body-mass index (BMI) in the Framingham Heart Study population (FHS), and height and seven metabolic syndrome traits (MetS) traits: BMI, waist-to-hip ratio (WHR), systolic blood pressure (SBP), fasting glucose (GLU), fasting insulin (INS), fasting triglycerides (TG), and fasting high-density lipoprotein (HDL) in the Atherosclerosis Risk in Communities population (ARIC) (ARIC MetS estimates shown in Table 1). Our base FHS population consisted of 4,240 subjects and our base ARIC population consisted of 8,451 subjects (see Methods and Tables S1 and S2 for a description of the populations). The genetic relationship between pairs of subjects was estimated using 436,126 genome-wide common SNP markers for ARIC and 320,118 SNPs for FHS (see Methods for details).

**Table 1:** *h^2^* and *h_g_^2^* estimates (ARIC population).

	BMI	WHR	GLU	INS	TG	HDL	SBP
h^2^	0.34 (0.12)	0.28 (0.12)	0.33 (0.12)	0.23 (0.12)	0.47 (0.11)	0.48 (0.11)	0.30 (0.12)
h_g_^2^	0.14 (0.05)	0.13 (0.05)	0.10 (0.05)	0.09 (0.05)	0.16 (0.05)	0.12 (0.05)	0.24 (0.05)

Mean and standard error estimates from univariate models.

We first estimated *h^2^* for related individuals with relationships between 0.35 and 0.65, derived empirically from the SNP markers, for height and BMI in the ARIC and FHS populations (see Methods for derivation of the relationship matrix). This resulted in 3,663 subjects (6,706,953 pairs of subjects) for FHS and 530 subjects (140,185 pairs of subjects) for ARIC. We found *h^2^* to be 0.77 (s.e. 0.03) for height and 0.39 (s.e. 0.04) for BMI in FHS, and 0.88 (s.e. 0.09) for height and 0.34 (s.e. 0.12) for BMI in ARIC. The estimated *h^2^* were consistent with values obtained using phenotypic regression (data not shown) and previous results [6], [7], [10], [11].

We then compared these values to estimates for *h_g_^2^* for unrelated individuals with relationships less than 0.025 (see Methods for derivation of the relationship matrix). This resulted in 1,489 subjects (1,107,816 pairs of subjects) for FHS and 5,647 subjects (31,882,962 pairs of subjects) for ARIC. As mentioned above, *h_g_^2^* provides an estimate of the heritability explained by common variants because of presumed lesser linkage disequilibrium between the common SNPs and the rest of the genome as compared to related individuals. We found *h_g_^2^* to be 0.50 (s.e. 0.18) for height and 0.10 (s.e. 0.18) for BMI in FHS, and 0.46 (s.e. 0.05) for height and 0.14 (s.e. 0.05) for BMI in ARIC. These values are consistent with previously estimated values [6], [7]. Using the average across FHS and ARIC estimates, this implied that the common SNPs accounted for approximately 58% of *h^2^* for height and 33% for BMI. To assess whether including more common SNPs would explain more of the *h^2^*, we examined how *h_g_^2^* depended on the number of SNPs. As shown in Figure S1, the mean and standard error of the *h_g_^2^* estimate for height in the ARIC population appeared to stabilize after approximately 300,000 SNPs.

We then estimated *h^2^* and *h_g_^2^* for the MetS traits in the ARIC population using the same subjects as above (see Table 1). We validated our *h^2^* estimates by using phenotypic regression between related individuals for some of the traits (data not shown). The median *h^2^* was 0.33, the minimum was 0.23 (INS), and the maximum was 0.48 (HDL). The median *h_g_^2^* was 0.13, the minimum was 0.09 (INS), and maximum was 0.24 (SBP). Comparing the medians suggested that *h_g_^2^* explains ∼39% of the *h^2^* for these MetS traits. We found that the common SNPs explained large proportions of the *h^2^*: 41% of *h^2^* for BMI, 46% for WHR, 30% for GLU, 39% for INS, 34% for TG, 25% for HDL, and 80% for SBP.

We next estimated the genetic correlations between MetS traits using a bivariate (multivariate) model (see Tables S3 and S4 for covariances). Table 2 shows the genetic and residual correlations for related individuals using bivariate models. The genetic correlation is the additive genetic covariance between traits normalized by the geometric mean of the individual trait genetic variances. The residual correlation is similarly estimated using the residual covariance and variances. For related individuals, we found significant genetic correlations for BMI-WHR, WHR-INS, GLU-INS, INS-TG, and TG-HDL and significant residual correlations between BMI-WHR, BMI-INS, BMI-HDL, WHR-INS, INS-HDL, and TG-HDL. Table 3 shows the genetic and residual correlations for the unrelated individuals. We found significant genetic correlations for BMI-WHR and TG-HDL and significant residual correlations for all of the estimates except SBP-HDL. The genetic correlations for unrelated individuals were proportional to the genetic correlations for related individuals (see Figure S2) with a proportionality constant of 0.44 (s.e. = 0.15 ; two-tail *t*-distribution *p*-value with 20 d.f. = 8.2*10^−3^). The phenotypic correlations between traits were similar for related and unrelated individuals and are shown in Table 4. These values were also consistent with the reported estimates in the National Heart Lung and Blood Institute-Family Heart Study (NHLBI-FHS), which included Framingham Heart Study and ARIC families [11].

**Table 2:** Genetic and residual correlation coefficients between MetS traits in the ARIC population among related individuals from the bivariate REML model.

	BMI	WHR	GLU	INS	TG	HDL	SBP
BMI		0.75 (0.16)*	0.23 (0.24)	0.17 (0.27)	0.19 (0.20)	−0.12 (0.21)	0.55 (0.24)
WHR	0.52 (0.08)*		0.35 (0.26)	0.67 (0.26)*	0.10 (0.22)	−0.12 (0.22)	0.37 (0.26)
GLU	0.19 (0.12)	0.14 (0.12)		0.69 (0.25)*	0.21 (0.21)	−0.07 (0.21)	0.13 (0.27)
INS	0.64 (0.08)*	0.35 (0.09)*	0.22 (0.11)		0.76 (0.21)*	−0.33 (0.23)	0.29 (0.29)
TG	0.29 (0.12)	0.34 (0.12)	0.21 (0.13)	0.27 (0.11)		−0.59 (0.13)*	0.21 (0.22)
HDL	−0.38 (0.12)*	−0.34 (0.12)	−0.22 (0.13)	−0.39 (0.11)*	−0.45 (0.11)*		−0.06 (0.23)
SBP	0.11 (0.12)	0.18 (0.11)	0.05 (0.12)	0.24 (0.11)	0.10 (0.13)	−0.02 (0.13)	

Mean and standard error of the Pearson correlation coefficient for genetic correlations (upper triangle) and residual correlations (lower triangle). An asterisk indicates significance with p<0.05 adjusted for 21 hypotheses using the two-tailed hypothesis test and normal distribution of the Fisher transformed correlation coefficient.

**Table 3:** Genetic and residual correlations between MetS traits in the ARIC population among unrelated individuals from the bivariate REML model.

	BMI	WHR	GLU	INS	TG	HDL	SBP
BMI		0.91 (0.18)*	0.01 (0.32)	0.57 (0.24)	0.20 (0.24)	−0.15 (0.28)	0.16 (0.20)
WHR	0.44 (0.03)*		0.09 (0.32)	0.33 (0.31)	0.32 (0.23)	−0.06 (0.30)	0.17 (0.21)
GLU	0.27 (0.04)*	0.18 (0.04)*		0.05 (0.40)	0.07 (0.30)	−0.16 (0.34)	0.11 (0.24)
INS	0.51 (0.03)*	0.40 (0.04)*	0.39 (0.04)*		0.22 (0.29)	−0.20 (0.36)	0.20 (0.25)
TG	0.31 (0.04)*	0.33 (0.04)*	0.20 (0.04)*	0.43 (0.04)*		−0.57 (0.19)*	0.002 (0.19)
HDL	−0.34 (0.04)*	−0.33 (0.04)*	−0.16 (0.04)*	−0.39 (0.04)*	−0.51 (0.03)*		−0.03 (0.22)
SBP	0.25 (0.05)*	0.18 (0.05)*	0.17 (0.05)*	0.22 (0.04)*	0.21 (0.05)*	−0.04 (0.05)	

Mean and standard error of the Pearson correlation coefficient for genetic correlations (upper triangle) and residual correlations (lower triangle). An asterisk indicates significance with p<0.05 adjusted for 21 hypotheses using the two-tailed hypothesis test and normal distribution of the Fisher transformed correlation coefficient.

**Table 4:** Phenotypic correlation coefficients between MetS traits in the ARIC population.

	BMI	WHR	GLU	INS	TG	HDL	SBP
BMI		0.59 (0.04)*	0.20 (0.04)*	0.49 (0.04)*	0.24 (0.04)*	−0.26 (0.04)*	0.25 (0.04)*
WHR	0.51 (0.01)*		0.21 (0.04)*	0.43 (0.04)*	0.23 (0.04)*	−0.24 (0.04)*	0.23 (0.04)*
GLU	0.24 (0.01)*	0.17 (0.01)*		0.34 (0.04)*	0.21 (0.04)*	−0.15 (0.04)*	0.07 (0.04)
INS	0.52 (0.01)*	0.39 (0.01)*	0.35 (0.01)*		0.42 (0.04)*	−0.35 (0.04)*	0.25 (0.04)*
TG	0.30 (0.01)*	0.33 (0.01)*	0.19 (0.01)*	0.40 (0.01)*		−0.52 (0.04)*	0.14 (0.04)*
HDL	−0.32 (0.01)*	−0.30 (0.01)*	−0.15 (0.01)*	−0.37 (0.01)*	−0.52 (0.01)*		−0.04 (0.04)
SBP	0.23 (0.01)*	0.18 (0.01)*	0.15 (0.01)*	0.21 (0.01)*	0.16 (0.01)*	−0.04 (0.01)*	

Mean and standard error of the Pearson correlation coefficient. Coefficients among related individuals shown in the upper triangle. Coefficients among unrelated individuals shown in the lower triangle. An asterisk indicates significance with p<0.05 adjusted for 21 hypotheses using the two-tailed hypothesis test and normal distribution of the Fisher transformed correlation coefficient.

We validated our genetic correlation estimates using bivariate models for each pair of traits by analyzing all 7 MetS traits simultaneously for the unrelated individuals in a single multivariate model. This 7 trait multivariate model was much more expensive computationally so we used a less stringent convergence rule. The results were similar to the bivariate model (see Table S5 and S6) although the genetic correlation increased and their error decreased for a number of the estimates. In addition to the significant genetic correlations in the bivariate models, we also found the genetic correlation for BMI-INS to be significant in the 7 trait model.

We then examined the relationship between the genetic and phenotypic correlations (see Figure S3). For related individuals, we found that the phenotypic correlations *r_p_* were proportional to the genetic correlations *r_g_* with a proportionality constant of 1.2 (s.e. = 0.16; two-tail *t*-distribution *p*-value with 20 d.f. = 3.1*10^−7^). For unrelated individuals, we found that the phenotypic correlations were proportional to the genetic correlations with a proportionality constant of 0.85 (s.e. = 0.19 ; two-tail *t*-distribution *p*-value with 20 d.f. = 2.3*10^−4^). The direct proportionality between *r_p_* and *r_g_* implies that the ratio *r_g_/r_p_* is approximately constant for the MetS traits.

## Discussion

We used a recently developed approach to analyzing GWAS data and provided new estimates for the total amount of additive genetic information contained in the common SNPs for MetS traits. The approach uses a linear mixed-effects model to estimate the additive genetic variances and correlations between traits. The model relies on knowing the genetic relationships between the individuals analyzed. Previously, this had been obtained from family pedigrees. Visscher et al. [9] and Yang et al. [6] observed that the genetic relationships could be computed from the GWAS SNPs. They also presumed that the heritability estimated for unrelated individuals with low SNP correlation are explained mainly by these common SNPs because the linkage disequilibrium between the common SNPs and the rest of the genome is weak. This would be in contrast to related individuals with high SNP correlation where linkage disequilibrium is strong. Thus, heritability estimated with the genetic relationships of unrelated individuals is attributed to the common SNPs while that estimated with the related individuals is attributed to the entire genome. This then creates a major distinction between *h^2^* and *h_g_^2^*. We computed both in the same population. However, differences between estimates of *h^2^* and *h_g_^2^* may also arise due to differences in environmental influences and non-additive genetic effects that may bias the estimates. Provided that these biases are small then the ratio of *h_g_^2^* to *h^2^* provides an estimate of the proportion of narrow sense heritability captured by the common SNPs.

We confirmed previous findings that a large proportion of *h^2^* is explained by the common SNPs. Our *h_g_^2^* estimates for height and BMI in two independent analyses (i.e. ARIC and FHS) were consistent with previously reported values [6], [7]. Our *h^2^* estimates for BMI, GLU, INS, TG, HDL, and SBP were similar to the findings of the large family National Heart, Lung, and Blood Institute (NHLBI) Family Heart Study [11], which included Framingham Heart Study and ARIC families. We found that *h_g_^2^* explained a large proportion of *h^2^* across the MetS traits, and *h_g_^2^* explained approximately 39% of the *h^2^* for these traits. We estimated that the common SNPs explain 58% of *h^2^* for height, 41% for BMI, 46% for WHR, 30% for GLU, 39% for INS, 34% for TG, 25% for HDL, and 80% for SBP. Our *h_g_^2^* findings are striking compared to traditional GWAS approaches where significant common SNPs have been shown to explain only 4% of *h^2^* for BMI with 32 SNPs, 11% for GLU with 14 SNPs, 20% for TG with 48 SNPs, 25% for HDL with 60 SNPs, 3% for SBP with 10 SNPs, and 12% for height with 180 SNPs [12]–[16]. Height had the largest absolute *h_g_^2^*, which was consistent with having a large *h^2^*. Surprisingly, SBP had the largest proportion of *h^2^* explained by the common SNPs while only a few percent of this has been uncovered by traditional GWAS. However, the standard error of *h_g_^2^* for SBP was large and reducing this error will be important for further investigation. Conversely, our analysis suggested that the SNP markers already identified for TG and HDL may contain the maximum heritability expected from the common SNPs.

Our analysis of *h_g_^2^* against the number of SNPs suggested that the mean and standard error of *h_g_^2^* for height is well estimated by approximately 300,000 markers and that including more markers would have little effect for this trait and perhaps others. The standard error of *h_g_^2^* also increased with SNP number. This may seem paradoxical but can be explained by recalling that the estimate for *h_g_^2^* is proportional to the regression coefficient of the square of the phenotype differences versus the genetic relationship (i.e. Haseman-Elston regression) [8]. The standard error of *h_g_^2^* is thus inversely proportional to the variance of the genetic relationship. Since the latter is estimated from the common SNPs, this variance is expected to decrease as the number of SNPs increases thereby increasing the standard error [6].

Using the bivariate (multivariate) model [17], [18] we estimated the genetic and residual correlations between the MetS traits. Among these, we found that the genetic correlations in related and unrelated individuals for BMI and WHR were significantly different from zero. This is consistent with both traits as indirect measures of body fat and common health risks [19]. Previously, Rice et al., 1994 [20] found significant genetic correlations between BMI and SBP among normotensive nonobese families. This suggested a common genetic etiology to their physiological relationship through hyperinsulinemia resulting in increased renal reabsorption of sodium and sympathetic activation [20]. We found a large genetic correlation among related subjects, although it was not significant because of the large error. This was consistent with the large family study by the NHLBI that did not find a significant genetic correlation [8]. Perusse et al, 1997 [21] argued that cross-trait resemblance between BMI and lipids is mostly environmental. In concordance, we did not find significant genetic correlations between either BMI or WHR and TG and HDL for either related or unrelated individuals (see Table 3 and Table 4) while residual (which includes environmental) correlations were significant for BMI–HDL. We found that the residual covariance accounted for a minimum of 71% (derived from the estimates in Table 4 and Table S3) of the phenotype covariance between BMI or WHR and the lipid measurements for related individuals. Genetic correlations between TG and HDL were also large, which is consistent with their direct physiological relationship [22]. This is also consistent with the findings from a recent GWAS meta-analysis whose results showed that 50% of the significant markers for TG were also significant for HDL (derived from Supplementary Tables 6 and 11 in [16]), and with a genome-wide LOD correlation analysis [23]. While we found some significant genetic correlations among both related and unrelated subjects, the variance was large for these estimates and greater statistical power is needed for better accuracy.

We found that the genetic correlation was directly proportional to the phenotypic correlation, which was an unexpected, empirical finding. Previously, a linear relationship between the correlations was hypothesized by Cheverud for sets of traits with common functions, and shown empirically for a number of traits [8], [24]–[26]. While this finding is interesting from an evolutionary genetics perspective, it may also serve a useful purpose in the maximum likelihood computation of the linear mixed-effects model by providing initial genetic correlation (i.e. covariance) estimates based on the phenotypic correlations.

In summary, we provided evidence that the common SNPs explain large proportions of the variance for several MetS traits in agreement with previous findings for some of these traits [6], [7]. This is consistent with the original premise of GWAS that a large proportion of phenotypic variation for common traits may be due to common variants [27]. However, an amendment to this premise is that it is likely to be many common variants with small effect. This is supported by recent meta-analyses with larger sample sizes that have identified more associated common SNPs. This approach can serve as a first approximation of the total heritability expected from common SNPs given a genome-wide set of markers and requires fewer subjects to achieve significant results. We also found genetic associations that will be useful for single gene and systems biology studies. Future studies with greater power will provide estimates for weaker multivariate genetic associations and provide greater precision for the estimates presented here.

## Methods

### ARIC population and GWAS data

Our main study population was the Atherosclerosis Risk In Communities (ARIC) population. The ARIC population consists of a large sample of unrelated individuals and some families across North America. The population was recruited from four centers across the United States: Forsyth County, North Carolina; Jackson, Mississippi; Minneapolis, Minnesota; and Washington County, Maryland. For this study, we restricted our analysis to the European-American group. The population was recruited in 1987 from the general population consisting of subjects aged 45 to 64 years. The ARIC population consisted of 8,451 subjects.

Quality control and genotype calls for common SNPs were evaluated previously for ARIC using the Affymetrix Human SNP Array 6.0. We selected bilallelic autosomal markers based on the following criteria: missingness <0.05, Hardy-Weinberg equilibrium (p<10^−6^) and minor allele frequency >0.05. Subjects with missingness >0.05 were removed. This resulted in 436,126 retained markers.

Quality control measurements from dbGAP (GENEVA ARIC Project Quality Control Report Sept 22, 2009) indicate significant population stratification between self-identified white (European-ancestory kind group) and black populations when projected onto HapMap components. Furthermore, principal-components analysis of the European-ancestory group by dbGAP showed that no component explained more than 0.1% of the population variance. For this study we only analyzed the European-ancestory group and treated it as a single population.

ARIC phenotypes were adjusted for age, sex, and study center. Only single measurements from visit 1 were used for these subjects. We only used subjects with negative diabetes status and with genotype and phenotype information for all traits. This resulted in 8,451 subjects. We standardized all the traits. We first log-transformed BMI, glucose, insulin, triglycerides, HDL, and systolic blood pressure. All laboratory measurements are under fasting conditions. Population trait statistics are in Table S1.

### Framingham Heart Study (FHS) population

We estimated *h^2^* and *h_g_^2^* for height and BMI in the Framingham Heart Study population (FHS). The FHS population is a large multi-generational dataset that started in 1948 in Framingham, Massachusetts in the United States. It consists of a number of ethnicities predominantly from the United Kingdom, Ireland, Italy, and Western Europe [28]. Markers were screened similarly to ARIC and we also removed any SNPs that did not overlap with the ARIC set, which results in 320,118 SNPs. We used principal components analysis of the linkage disequilibrium (LD) pruned genetic relationship matrix to identify components with variance >0.1%. LD pruning was as in the ARIC 2009 report. This resulted in 73,432 retained SNPs. We found three significant components that were then used as covariates in the REML model. For consistency with ARIC, we restricted the age range at time of exam to 45 to 65 years and randomly selected a single measurement in the case of multiple measurements. Phenotypes were adjusted for age, sex, and generation prior to the REML estimation and standardized. We first log-transformed BMI. Population trait statistics are in Table S2. Our base FHS population consisted of 4,240 subjects.

### *h* ^2^ estimates using common SNP estimated relationship

We determined *h^2^* using the linear mixed-effects model (see derivation below) and related individuals defined as genomic relatedness between 0.35 and 0.65. We assume that the common SNPs are in greater linkage disequilbrium among related individuals and, as such, can be used to estimate the total additive-genetic variance across the allele spectrum as suggested by Visscher et al., 2006 [9]. We constrained the relationship matrix to have at least one related pair per subject. This was done by pruning the entire population relationship matrix by randomly selecting a row and removing the row and its corresponding column if no genomic covariance in the row was between the cutoff values. For all pairs, including unrelated individuals, we used their empirically defined relationship. This resulted in 530 individuals being selected for analysis in ARIC and 3,663 individuals in FHS.

*h^2^* was estimated with *h^2^* = *var_g_*/(*var_g_*+*var_e_*), where *var_g_* and *var_e_* are the genetic and residual variance components estimated by the REML model using related individuals. The error was estimated from the inverse Fisher Information (see linear mixed-effects model below) and propagated using a first-order Taylor expansion.

### Common SNP linear mixed-effects model estimate of *h*_g_^2^

We used the linear mixed-effects model and only unrelated individuals to estimate the additive-genetic variance attributable to the common SNPs (*h_g_^2^*). Unrelated individuals were defined as subjects with maximum genomic correlation of <0.025. The genomic relationship matrix was then produced as above based on this cutoff. The cutoff was taken from Yang et al. 2010 [6] and is less than the expected coefficient of relatedness between 2^nd^ cousins. For these estimates we used the same group of 5,647 unrelated individuals for all estimates in ARIC and 1,489 individuals in FHS. *h_g_^2^* was estimated as *h_g_^2^* = *var_g_*/(*var_g_*+*var_e_*), where *var_g_* and *var_e_* are the genetic and residual variance components estimated by the REML model using unrelated individuals. The standard error was estimated as above. The height h_g_^2^ versus SNP number analyses were performed over allele frequency range of 0.05 to 0.5 in order of increasing and decreasing frequency.

### Correlations

The genetic correlation (*r_g_*) is defined as 
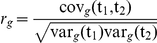
, where (var_g_(*t_i_*)) is the additive genetic variance of trait *i* and covariance (cov_g_(*t_i_,t_j_*)) is the additive genetic covariance between the traits. The variances and covariances are estimated directly in the multivariate linear mixed-effects model. The error was computed from the estimated errors of the variances and covariance using a first-order Taylor expansion. The residual and phenotypic correlations were analogously defined. Phenotype correlations and error were estimated by linear regression of the standardized phenotypes.

### Proportionality constants

The mean and errors for proportionality constants between the genetic and phenotypic correlations were determined by randomly sampling over the distributions of the parameter estimates (i.e. Monte Carlo method) assuming that the error around the mean parameter estimate was normally distributed and that the parameters were independent. We then fit a linear function with the y-intercept fixed at 0 (after first confirming that it was not significantly different from zero).

### Significance testing

We assessed significance for correlation coefficients (*r*) using the standardized Fisher transformed estimate of r: arctan(*r*)/arctan(s.e.(r)). We estimated the two-tailed p-value from a normal distribution and significance was determined by p<0.05 and Bonferroni corrected for 21 hypotheses.

Significance for regression coefficient (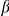) was estimated using the standardized coefficient 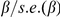. We estimated the two-tailed p-value from a t-distribution and 20 degrees of freedom and significance was determined by p<0.05.

Preprocessing of SNPs and phenotypes was done using PLINK [29] (v1.07,http://pngu.mgh.harvard.edu/purcell/plink/) and MATLAB (2010b, MathWorks, Natick, MA). REML optimization was executed using software written in MATLAB.

### Bivariate (multivariate) linear mixed-effects linear model

We considered the following multivariate linear mixed-effects model for *m* individuals, *n* loci and *t* traits [6]–[8], [17], [18], [30]:
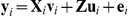
where **y** *_i_* is a *m*×1 vector of trait *i* for m individuals, **X**_i_ is an *m*×*s* fixed effects matrix for trait *i*, **v** *_i_* is a *s*×1 vector of fixed effects parameters for trait *i*, **Z** is an *m*×*n* matrix of standardized genotypes, **u** *_i_* is an *n*×1 vector of random effects for trait *i* satisfying **u**_i_∼**N**(**0**,**G**) and **e** *_i_* is an *m*×1 vector of residual effects satisfying **e**_i_∼**N**(**0**,**R**), with matrix blocks **G**_ij_ = cov_gij_ **I**_n_ and **R**_ij_ = cov_eij_ **I**_m_ and **I** *_l_* is the *l*×*l* identity matrix. This model can be used for single or multiple traits. For two traits, it is called a bivariate model. The model is identical to that used by [6], [7], [17].

We considered only bi-allelic SNPs in Hardy-Weinberg equilibrium. Denote the minor allele by q and the major allele by Q. Let the minor allele frequency at locus *i* have frequency *p*_i_. We assign a value of 2 for genotype qq, 1 for genotype qQ and 0 for genotype QQ. The Hardy-Weinberg mean frequency for the genotype at locus *i* is 2*p*_i_ and the variance is 2*p_i_*(1−*p_i_*). The standardized genotype entries have values of (2−2*p*_i_)/(2*p*_i_(1−2*p*_i_))^1/2^ for qq, (1−2*p*_i_)/(2*p*_i_(1−2*p*_i_))^1/2^ for qQ, and −2*p*_i_/(2*p*_i_(1−2*p*_i_))^1/2^ for the QQ genotype.

The log of the likelihood function is given by
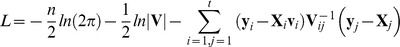
where the covariance matrix can be expressed as a tensor product 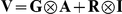 with *m*×*m* blocks **V** ^−1^ *_ij_* and **A** is the genetic relationship matrix. Following Yang et al. [6], we used a modified covariance matrix for **A**, 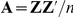, where the diagonals of **A** are computed using the formula
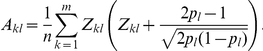
We use the restricted maximum likelihood (REML) approach [8] where the gradients of the log likelihood are given by
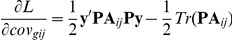


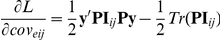
where **I** *_ij_* is a *tm*×*tm* dimensional matrix with zero entries except for a *m*×*m* identity matrix at block location *i*, *j*, 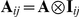 and 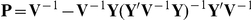, where 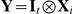.

We solved the REML equations using an EM algorithm [8], which was given by
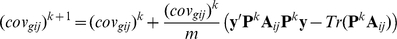


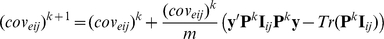
for iteration *k*+1 in terms of iteration *k*. We iterated until the rate of change of the log likelihood function was less than about 10^−4^. We also checked that the rate of change of the square of the covariance predictions was less than 10^−8^. We checked our results against the software developed by Yang et al. (GCTA) [31] for the univariate model.

For the multivariate model, we transformed to a coordinate system where the covariance matrices were diagonal [8] to speed up the computation. Let **z**_j_ be the set of phenotypes for individual *j*. We used the canonical transformation 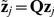 such that 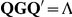 and 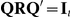. **Q** can be computed from the formula 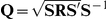 where 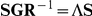, (**S** is the matrix of left eigenvectors of **GR** ^−1^). The transformed genetic covariances are given by 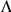 and the residual covariances are **I** *_t_*. Each step consisted of taking a single step with the univariate EM algorithm for the transformed additive genetic and residual variance followed by a transformation back to the original coordinates. We iterated until the maximum of the magnitudes of the components of the gradient of the log likelihood function was less than approximately 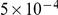.

In our computations, we used both the direct EM algorithm and the canonically transformed algorithm because even though the transformed algorithm was in principle faster, it sometimes had poor convergence properties if the initial guess was not sufficiently close to the maximum likelihood value. We ensured that both give the same results. For computational efficiency, the results shown are computed from the bivariate model for the different trait pairs. We confirmed our results with a multivariate model that included all traits.

Our error estimates were given by the inverse of the Fisher information matrix **F**, which we computed by evaluating the Hessian of the log likelihood at the maximum likelihood predictions. **F** is a *t(t+1)*×*t(t+1)* dimensional matrix with rows corresponding to the genetic and residual variances and covariances (where cov_ij_ was set equal to cov_ji_) and with block elements (that are not all contiguous) given by
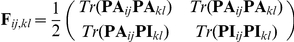
for 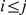
and 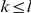.

## Supporting Information

Figure S1 Height *h*_g_^2^ versus number of SNPs by sampling the allele frequency from 0.05 to 0.5 (red = low to high, blue = high to low, black = using all SNPs). A) *h*_g_^2^ estimates for height relative to the number of SNPs (mean and s.e.). B) Standard error versus number of SNPs.
(TIF)

Figure S2 Genetic correlation coefficient for unrelated individuals versus the genetic correlation coefficients for related individuals. Shown are the mean and standard errors. Dashed line is the least squares fit with the y-intercept fixed at 0 estimated using a Monte Carlo method (slope = 0.44).
(TIF)

Figure S3 A) Genetic correlation coefficients versus the phenotypic correlation coefficients for related individuals. Shown are the mean and standard errors. Dashed line is the least squares fit with the y-intercept fixed at 0 estimated using a Monte Carlo method (slope = 1.2). B) Genetic correlation coefficients versus the phenotype correlation coefficients for unrelated individuals. Shown are the mean and standard errors. Dashed line is the least squares fit with the y-intercept fixed at 0 estimated using a Monte Carlo method (slope = 0.85).
(TIF)

Table S1 Atherosclerosis Risk in Communities Study (ARIC) population statistics by sex; mean (sd; minimum-maximum). BMI = body-mass index, WC = waist circumference, WHR = waist-to-hip ratio, GLU = fasting glucose, INS = fasting insulin, TG = fasting triglycerides, HDL = fasting high-density lipoprotein, SBP = systolic blood pressure.
(DOCX)

Table S2 Framingham Heart Study (FHS) population statistics.
(DOCX)

Table S3 Genetic and residual covariance estimates for the ARIC population among related individuals. Mean and standard error of genetic (upper triangle) and residual (lower triangle) covariance estimates from the univariate (diagonals) and bivariate (off-diagonals) REML model.
(DOCX)

Table S4 Genetic and residual covariance estimates for the ARIC population among unrelated individuals. Mean and standard error of genetic (upper triangle) and residual (lower triangle) covariance estimates from the univariate (diagonals) and bivariate (off-diagonals) REML model.
(DOCX)

Table S5 Genetic (upper triangle) and residual (lower triangle) correlations among unrelated individuals in the ARIC population based on simultaneous analysis of all MetS traits. Mean and standard error of the Pearson correlation coefficient for genetic correlations (upper triangle) and residual correlations (lower triangle). An asterisk indicates significance with p<0.05 adjusted for 21 hypotheses using the two-tailed hypothesis test and normal distribution of the Fisher transformed correlation coefficient.
(DOCX)

Table S6 Genetic (upper triangle) and residual (lower triangle) covariances among unrelated individuals in the ARIC population based on simultaneous analysis of all MetS traits. Mean and standard error.
(DOCX)
