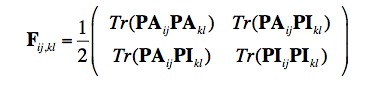# Correction: Heritability and Genetic Correlations Explained by Common SNPs for Metabolic Syndrome Traits

**DOI:** 10.1371/annotation/61bb5924-6688-4ee5-a37f-d48aa09ad66a

**Published:** 2012-06-14

**Authors:** Shashaank Vattikuti, Juen Guo, Carson C. Chow

In the Methods section, there are errors in the second, sixth, seventh and eighth equations in the eighth rule of the sub-section titled "Bivariate (multivariate) linear mixed-effects linear model".

- The current second equation in this sub-section is incorrect and should appear as: 


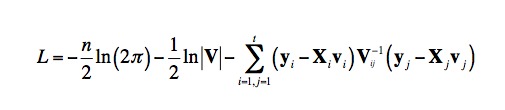


- The current sixth equation in this sub-section is incorrect and should appear as: 


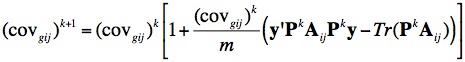


- The current seventh equation in this sub-section is incorrect and should appear as: 


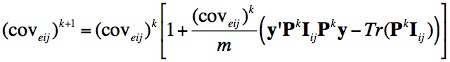


- The current eighth equation in this sub-section is incorrect and should appear as: