# Correction: Heritability and Genetic Correlations Explained by Common SNPs for Metabolic Syndrome Traits

**DOI:** 10.1371/annotation/8f3f1f56-6731-486e-8d55-5fa09b476cad

**Published:** 2012-06-14

**Authors:** Shashaank Vattikuti, Juen Guo, Carson C. Chow

In the Methods section, there was an error in the equation in the last sentence of the second paragraph in the subsection titled "Bivariate (multivariate) linear mixed-effects linear model." Please view the complete, correct equation here: http://www.plosgenetics.org/corrections/pgen.1002637_Method_Error.cn.tiff

