# Supplementary figures and images for: Heritability and Genetic Correlations Explained by Common SNPs for Metabolic Syndrome Traits
Source: PLoS Genet. 2012 Mar 29;8(3):e1002637. doi: 10.1371/journal.pgen.1002637 (PMC3315484; doi:10.1371/journal.pgen.1002637)

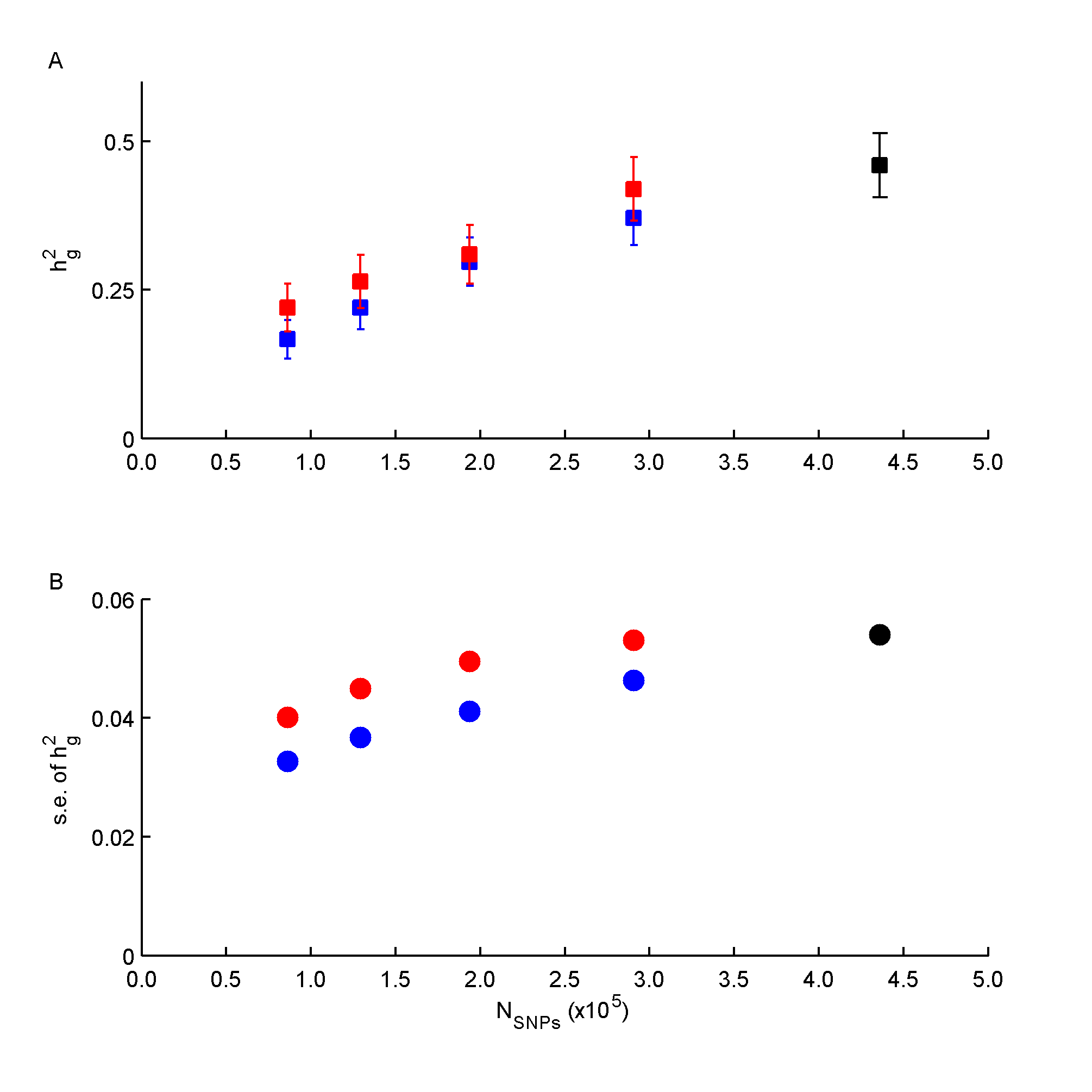

Supplement: Figure S1 — Height h g 2 versus number of SNPs by sampling the allele frequency from 0.05 to 0.5 (red = low to high, blue = high to low, black = using all SNPs). A) h g 2 estimates for height relative to the number of SNPs (mean and s.e.). B) Standard error versus number of SNPs. (TIF) [file pgen.1002637.s001.tif]

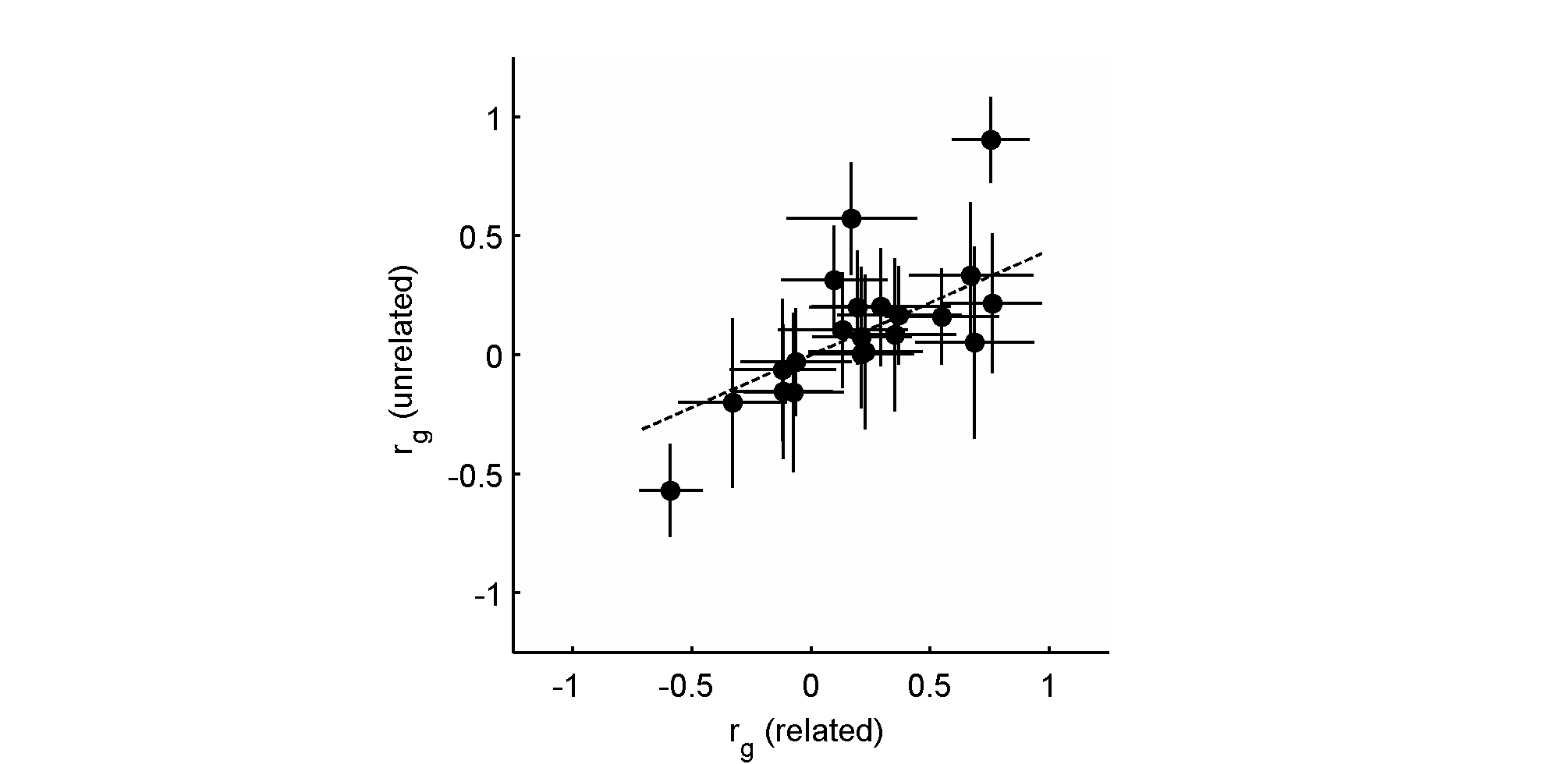

Supplement: Figure S2 — Genetic correlation coefficient for unrelated individuals versus the genetic correlation coefficients for related individuals. Shown are the mean and standard errors. Dashed line is the least squares fit with the y-intercept fixed at 0 estimated using a Monte Carlo method (slope = 0.44). (TIF) [file pgen.1002637.s002.tif]

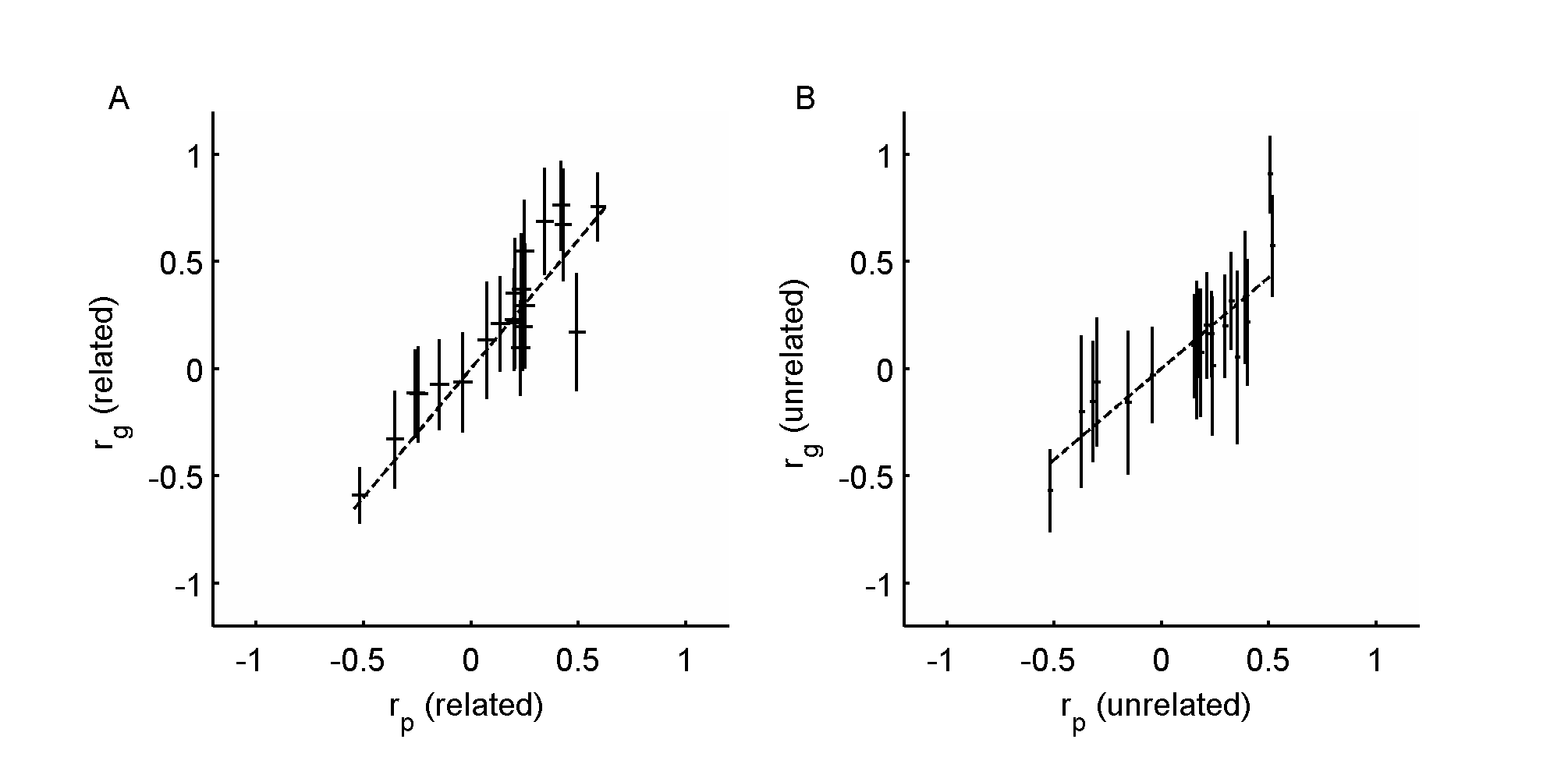

Supplement: Figure S3 — A) Genetic correlation coefficients versus the phenotypic correlation coefficients for related individuals. Shown are the mean and standard errors. Dashed line is the least squares fit with the y-intercept fixed at 0 estimated using a Monte Carlo method (slope = 1.2). B) Genetic correlation coefficients versus the phenotype correlation coefficients for unrelated individuals. Shown are the mean and standard errors. Dashed line is the least squares fit with the y-intercept fixed at 0 estimated using a Monte Carlo method (slope = 0.85). (TIF) [file pgen.1002637.s003.tif]
