# Supplementary material for: Heritability and Genetic Correlations Explained by Common SNPs for Metabolic Syndrome Traits
Source: PLoS Genet. 2012 Mar 29;8(3):e1002637. doi: 10.1371/journal.pgen.1002637 (PMC3315484; doi:10.1371/journal.pgen.1002637)
Supplement: Table S1 — Atherosclerosis Risk in Communities Study (ARIC) population statistics by sex; mean (sd; minimum-maximum). BMI = body-mass index, WC = waist circumference, WHR = waist-to-hip ratio, GLU = fasting glucose, INS = fasting insulin, TG = fasting triglycerides, HDL = fasting high-density lipoprotein, SBP = systolic blood pressure. (DOCX) [file pgen.1002637.s004.docx]

Table S1. Atherosclerosis Risk in Communities Study (ARIC) population

statistics by sex; mean (sd; minimum-maximum)

| Sex | M | F |
| --- | --- | --- |
| Number of subjects | 3,906 | 4,545 |
| Age, yrs | 55  (5.6; 44-66) | 54  (5.7; 44-65) |
| Height, m | 1.8  (0.06;1.4-2.0) | 1.6  (0.06; 1.4-1.8) |
| BMI, kg/m^2^ | 27  (3.9; 16-56) | 26  (5.2;14-53) |
| WHR | 0.97  (0.052; 0.62-1.39) | 0.89  (0.078; 0.49-1.29) |
| GLU, mM | 5.6  (0.49; 3.4-6.9) | 5.4  (0.49; 3.5-6.9) |
| INS, pM | 79  (55.2; 7.2-925.6) | 67  (49.8; 7.2-825.1) |
| TG, mM | 1.6  (1.0; 0.27-21.2) | 1.4  (0.78; 0.29-17.6) |
| HDL, mM | 1.1  (0.32; 0.25-3.3) | 1.5  (0.44; 0.35-3.5) |
| SBP, mmHg | 119  (15.7; 61-203) | 116  (17.2; 72-206) |

BMI=body-mass index, WC=waist circumference, WHR=waist-to-hip ratio, GLU=fasting glucose, INS=fasting insulin, TG=fasting triglycerides, HDL=fasting high-density lipoprotein, SBP=systolic blood pressure.
