# Supplementary material for: Heritability and Genetic Correlations Explained by Common SNPs for Metabolic Syndrome Traits
Source: PLoS Genet. 2012 Mar 29;8(3):e1002637. doi: 10.1371/journal.pgen.1002637 (PMC3315484; doi:10.1371/journal.pgen.1002637)
Supplement: Table S2 — Framingham Heart Study (FHS) population statistics. (DOCX) [file pgen.1002637.s005.docx]

Table S2. Framingham Heart Study (FHS) population statistics

| N_subjects | 4240 |
| --- | --- |
| Male/Female | 1916/2324 |
| Age (yrs) | Mean 55 (s.d. 6) |
| Generation 0 | 949 |
| Generation 1 | 3291 |
| BMI (kg/m^2^) | Mean 27 (s.d. 5) |
| Height (m) | Mean 1.7 (s.d. 0.09) |
