# Supplementary material for: Heritability and Genetic Correlations Explained by Common SNPs for Metabolic Syndrome Traits
Source: PLoS Genet. 2012 Mar 29;8(3):e1002637. doi: 10.1371/journal.pgen.1002637 (PMC3315484; doi:10.1371/journal.pgen.1002637)
Supplement: Table S3 — Genetic and residual covariance estimates for the ARIC population among related individuals. Mean and standard error of genetic (upper triangle) and residual (lower triangle) covariance estimates from the univariate (diagonals) and bivariate (off-diagonals) REML model. (DOCX) [file pgen.1002637.s006.docx]

Table S3. Genetic and residual covariance estimates for the ARIC population among related individuals.

|  |  | BMI | WHR | GLU | INS | TG | HDL | SBP |  |
| --- | --- | --- | --- | --- | --- | --- | --- | --- | --- |
|  |  | 0.34 (0.12) | 0.23 (0.10) | 0.08 (0.09) | 0.05 (0.10) | 0.08 (0.09) | -0.05 (0.09) | 0.18 (0.09) | BMI |
| BMI | 0.66 (0.11) |  | 0.28 (0.12) | 0.11 (0.09) | 0.16 (0.09) | 0.04 (0.09) | -0.05 (0.09) | 0.11 (0.09) | WHR |
| WHR | 0.37 (0.09) | 0.72 (0.12) |  | 0.33 (0.12) | 0.19 (0.09) | 0.08 (0.09) | -0.03 (0.09) | 0.04 (0.09) | GLU |
| GLU | 0.13 (0.08) | 0.10 (0.08) | 0.67 (0.12) |  | 0.23 (0.12) | 0.25 (0.09) | -0.11 (0.09) | 0.08 (0.09) | INS |
| INS | 0.45 (0.09) | 0.27 (0.09) | 0.15 (0.09) | 0.78 (0.12) |  | 0.47 (0.12) | -0.27 (0.10) | 0.08 (0.09) | TG |
| TG | 0.17 (0.08) | 0.20 (0.08) | 0.13 (0.08) | 0.17 (0.09) | 0.53 (0.11) |  | 0.48 (0.12) | -0.02 (0.09) | HDL |
| HDL | -0.22 (0.08) | -0.21 (0.08) | -0.13 (0.08) | -0.25 (0.09) | -0.24 (0.09) | 0.53 (0.11) |  | 0.31 (0.12) | SBP |
| SBP | 0.08 (0.08) | 0.13 (0.08) | 0.03 (0.08) | 0.18 (0.09) | 0.06 (0.08) | -0.01 (0.08) | 0.70 (0.12) |  |  |
|  | BMI | WHR | GLU | INS | TG | HDL | SBP |  |  |

Mean and standard error of genetic (upper triangle) and residual (lower triangle) covariance estimates from the bivariate linear mixed-effects model.
