# Supplementary material for: Heritability and Genetic Correlations Explained by Common SNPs for Metabolic Syndrome Traits
Source: PLoS Genet. 2012 Mar 29;8(3):e1002637. doi: 10.1371/journal.pgen.1002637 (PMC3315484; doi:10.1371/journal.pgen.1002637)
Supplement: Table S5 — Genetic (upper triangle) and residual (lower triangle) correlations among unrelated individuals in the ARIC population based on simultaneous analysis of all MetS traits. Mean and standard error of the Pearson correlation coefficient for genetic correlations (upper triangle) and residual correlations (lower triangle). An asterisk indicates significance with p<0.05 adjusted for 21 hypotheses using the two-tailed hypothesis test and normal distribution of the Fisher transformed correlation coefficient. (DOCX) [file pgen.1002637.s008.docx]

Table S5. Genetic and residual correlations between MetS traits in the ARIC population among unrelated individuals based on simultaneous analysis of all traits.

|  | BMI | WHR | GLU | INS | TG | HDL | SBP |
| --- | --- | --- | --- | --- | --- | --- | --- |
| BMI |  | 0.90 (0.16)* | 0.18 (0.25) | 0.61 (0.19)* | 0.30 (0.20) | -0.23 (0.24) | 0.13 (0.19) |
| WHR | 0.43 (0.04)* |  | 0.16 (0.25) | 0.46 (0.22) | 0.37 (0.19) | -0.14 (0.25) | 0.18 (0.19) |
| GLU | 0.25 (0.04)* | 0.17 (0.04)* |  | 0.40 (0.25) | 0.24 (0.23) | -0.24 (0.27) | 0.04 (0.21) |
| INS | 0.50 (0.03)* | 0.38 (0.04)* | 0.35 (0.04)* |  | 0.32 (0.22) | -0.34 (0.26) | 0.15 (0.21) |
| TG | 0.30 (0.04)* | 0.32 (0.04)* | 0.18 (0.04)* | 0.42 (0.04)* |  | -0.61 (0.16)* | 0.06 (0.18) |
| HDL | -0.34 (0.04)* | -0.34 (0.04)* | -0.14 (0.04)* | -0.50 (0.03)* |  |  | -0.02 (0.21) |
| SBP | 0.26 (0.05)* | 0.17 (0.05)* | 0.18 (0.05)* | 0.23 (0.05)* | 0.20 (0.05)* | -0.05 (0.05)* |  |

Mean and standard error of the Pearson correlation coefficient for genetic correlations (upper triangle) and residual correlations (lower triangle). An asterisk indicates significance with p<0.05 adjusted for 21 hypotheses using the two-tailed hypothesis test and normal distribution of the Fisher transformed correlation coefficient.
