# Supplementary material for: Heritability and Genetic Correlations Explained by Common SNPs for Metabolic Syndrome Traits
Source: PLoS Genet. 2012 Mar 29;8(3):e1002637. doi: 10.1371/journal.pgen.1002637 (PMC3315484; doi:10.1371/journal.pgen.1002637)
Supplement: Table S6 — Genetic (upper triangle) and residual (lower triangle) covariances among unrelated individuals in the ARIC population based on simultaneous analysis of all MetS traits. Mean and standard error. (DOCX) [file pgen.1002637.s009.docx]

Table S6. Genetic and residual covariance estimates for the ARIC cohort among unrelated individuals based on 7-trait linear mixed-effects model.

|  |  | BMI | WHR | GLU | INS | TG | HDL | SBP |  |
| --- | --- | --- | --- | --- | --- | --- | --- | --- | --- |
|  |  | 0.16 (0.05) | 0.14 (0.04) | 0.03 (0.04) | 0.09 (0.04) | 0.05 (0.04) | -0.03 (0.04) | 0.03 (0.04) | BMI |
| BMI | 0.84 (0.05) |  | 0.16 (0.05) | 0.02 (0.04) | 0.07 (0.04) | 0.07 (0.04) | -0.02 (0.04) | 0.04 (0.04) | WHR |
| WHR | 0.37 (0.09) | 0.84 (0.05) |  | 0.13 (0.05) | 0.05 (0.04) | 0.04 (0.04) | -0.03 (0.04) | 0.01 (0.04) | GLU |
| GLU | 0.21 (0.08) | 0.14 (0.08) | 0.87 (0.06) |  | 0.13 (0.05) | 0.05 (0.04) | -0.04 (0.04) | 0.03 (0.04) | INS |
| INS | 0.43 (0.09) | 0.33 (0.08) | 0.30 (0.08) | 0.88 (0.06) |  | 0.19 (0.05) | -0.10 (0.04) | 0.01 (0.04) | TG |
| TG | 0.24 (0.08) | 0.26 (0.08) | 0.15 (0.08) | 0.35 (0.08) | 0.81 (0.05) |  | 0.14 (0.05) | 0.00 (0.04) | HDL |
| HDL | -0.29 (0.08) | -0.28 (0.08) | -0.12 (0.08) | -0.33 (0.08) | -0.42 (0.09) | 0.87 (0.05) |  | 0.24 (0.06) | SBP |
| SBP | 0.21 (0.08) | 0.14 (0.08) | 0.15 (0.08) | 0.19 (0.08) | 0.15 (0.08) | -0.04 (0.08) | 0.76 (0.05) |  |  |
|  | BMI | WHR | GLU | INS | TG | HDL | SBP |  |  |
